# Yolk sac tumor differentiation in urothelial carcinoma of the urinary bladder: a case report and differential diagnosis

**DOI:** 10.1186/s13000-020-00983-3

**Published:** 2020-06-03

**Authors:** Nadia Espejo-Herrera, Enric Condom-Mundó

**Affiliations:** Department of Pathology, Hospital Universitari de Bellvitge, [Bellvitge Biomedical Research Institute] IDIBELL, Feixa Llarga S/N., 08907. L’Hospitalet de Llobregat, Barcelona, Spain

**Keywords:** Yolk sac tumor differentiation, Urothelial carcinoma, Urinary bladder, Case report

## Abstract

**Background:**

Yolk sac tumor (YST) is a germ cell neoplasm that arises predominantly in the gonads, but can also derive from somatic neoplasms in extragonadal locations. These latter cases have been documented in several organs, although reports from the urinary tract are limited. To our knowledge, this is the first report of a bladder urothelial carcinoma with a predominant component of YST differentiation.

**Case presentation:**

We present a unique case of a 76-year-old man with a recurrent urinary bladder tumor, initially interpreted as a high grade urothelial carcinoma with glandular differentiation. In the recurrent tumor, diverse histological patterns were identified, including glandular, hepatoid and sarcomatoid. This tumor showed positivity for AFP, GLP3 and SALL4, and negativity for CK7 and EMA. Fluorescent in situ hybridization study showed a polysomic pattern of chromosome 12. All these findings led to the final diagnosis of a YST derived from urothelial carcinoma.

**Conclusions:**

YST differentiation should be considered in the differential diagnosis of a high grade urothelial carcinoma, particularly when glandular and other unusual patterns are observed.

## Background

Yolk sac tumor (YST) is a germ cell neoplasm that arises predominantly within the gonads, but a significant minority of cases can be found in extragonadal midline locations, such as sacrococcygeal region, mediastinum, retroperitoneum and brain [1]. By the other hand, diverse somatic neoplasms may present areas of YST differentiation. Some authors denominated this group of neoplasms as “somatically derived YSTs” (SD-YSTs), and several cases have been reported, mainly from gastrointestinal and gynecologic sites [2]. In contrast, cases of urothelial carcinoma with YST features are scarce in the literature, and among those cases, YST differentiation has not been confirmed with additional studies [3, 4]. Herein, we present a case of a recurrent urinary bladder neoplasm, initially diagnosed as urothelial carcinoma with glandular differentiation, which showed multiple unusual histological patterns, and raised the diagnostic possibility of a YST differentiation. This report aims to increase the limited literature available for YSTs derived from urothelial neoplasms.

## Case presentation

**Fig. 1:**
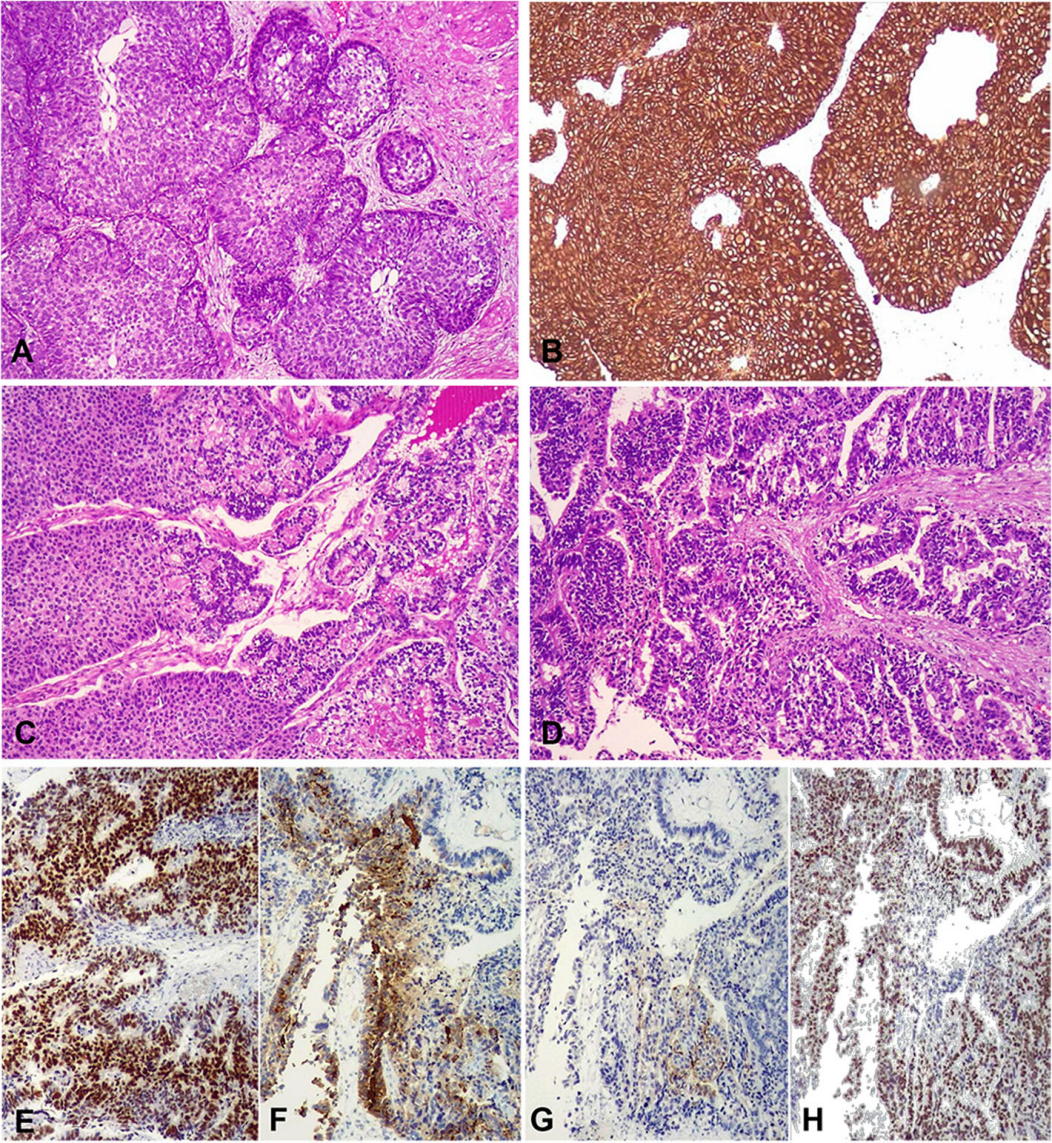
Surgical specimen of previous nephroureterectomy exhibiting a high grade urothelial carcinoma with conventional pattern (HE) (**a**) positive for CK7 (**b**). Interphase between conventional urothelial carcinoma and glandular pattern (HE) **(c)**. Areas with glandular pattern (HE) (**d**). CDX2 (**e**). AFP (**f**). GLP3 (**g**). SALL4 (**h**) in areas with glandular pattern

A 76-year-old man with previous history of smoking, type 2 diabetes mellitus, dyslipidemia, high blood pressure and alkaptonuria with ochronotic arthropathy, was referred to our hospital by edema in lower extremities and renal dysfunction. Image studies showed a papillary proliferation in the distal portion of the right ureter. The biopsy of this lesion revealed a high grade urothelial carcinoma **(**Fig. 1**)**, and the patient was treated with a right nephroureterectomy. One year later, the tumor relapsed and the patient underwent a transurethral resection (TUR) of a lesion located in the right wall of the bladder. This sample was diagnosed as a high grade urothelial carcinoma with extensive glandular differentiation. During the subsequent year, a positron emission tomography (^18^F-FDG PET/CT) showed areas of intense uptake in the urinary bladder, in multiple pelvic lymph nodes and in a mass located in the right thoracic wall, where a surgical trocar was placed during the previous nephroureterectomy. The patient was submitted to radical cystectomy, pelvic lymphadenectomy and excision of the thoracic mass.

**Fig. 2:**
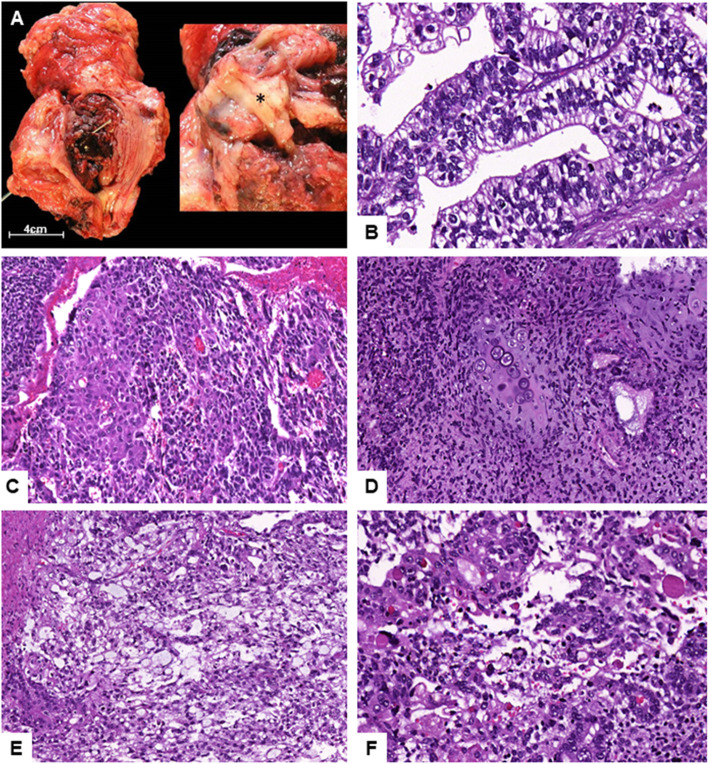
Surgical specimen of cystectomy showing a macroscopically heterogeneous tumor. Solid areas are marked with*(**a**). Microscopically the tumor exhibited an area of glandular pattern with supra and infranuclear vacuoles (**b**). Area with hepatoid pattern (**c**). Area with an undifferentiated sarcomatoid component and focus of chondroid differentiation (HE) (**d**). Areas with reticular-microcystic pattern exhibiting hyaline globules (HE) (**e-f**)

The specimen of cystectomy showed a polypoid tumor of 10 cm diameter filling the lumen of the bladder. The tumor was implanted on the right wall, involved the right ureteral meatus and grossly extended into the paravesical adipose tissue. Macroscopically, the lesion was heterogeneous, with extensive necrotic and hemorrhagic areas. Solid areas with white-tan color were also identified **(**Fig. 2a**)**. Microscopically, the tumor exhibited several components and patterns. First, an epithelial component, organized in papillary and glandular structures with tall columnar cells. These cells had clear cytoplasm and showed infra and supranuclear vacuoles, resembling endometrium or primitive intestinal epithelium. Second, an epithelial component organized in trabecular and solid patterns composed of polygonal cells with wide eosinophilic cytoplasm, central nuclei and nucleoli, similar to hepatocytes. Third, an undifferentiated stromal component with densely cellular areas composed of round, blue and small cells, intermingled with less cellular areas with myxoid stroma and nodules of chondroid tissue with atypical features. Fourth, an epithelial component with reticular and microcystic patterns, composed of atypical cells exhibiting intra and extracellular hyaline globules **(**Fig. 2b-f**)**. All these components were admixed. Only a focal component of conventional urothelial carcinoma was identified in the neighboring area of the right ureteral meatus.

**Fig. 3:**
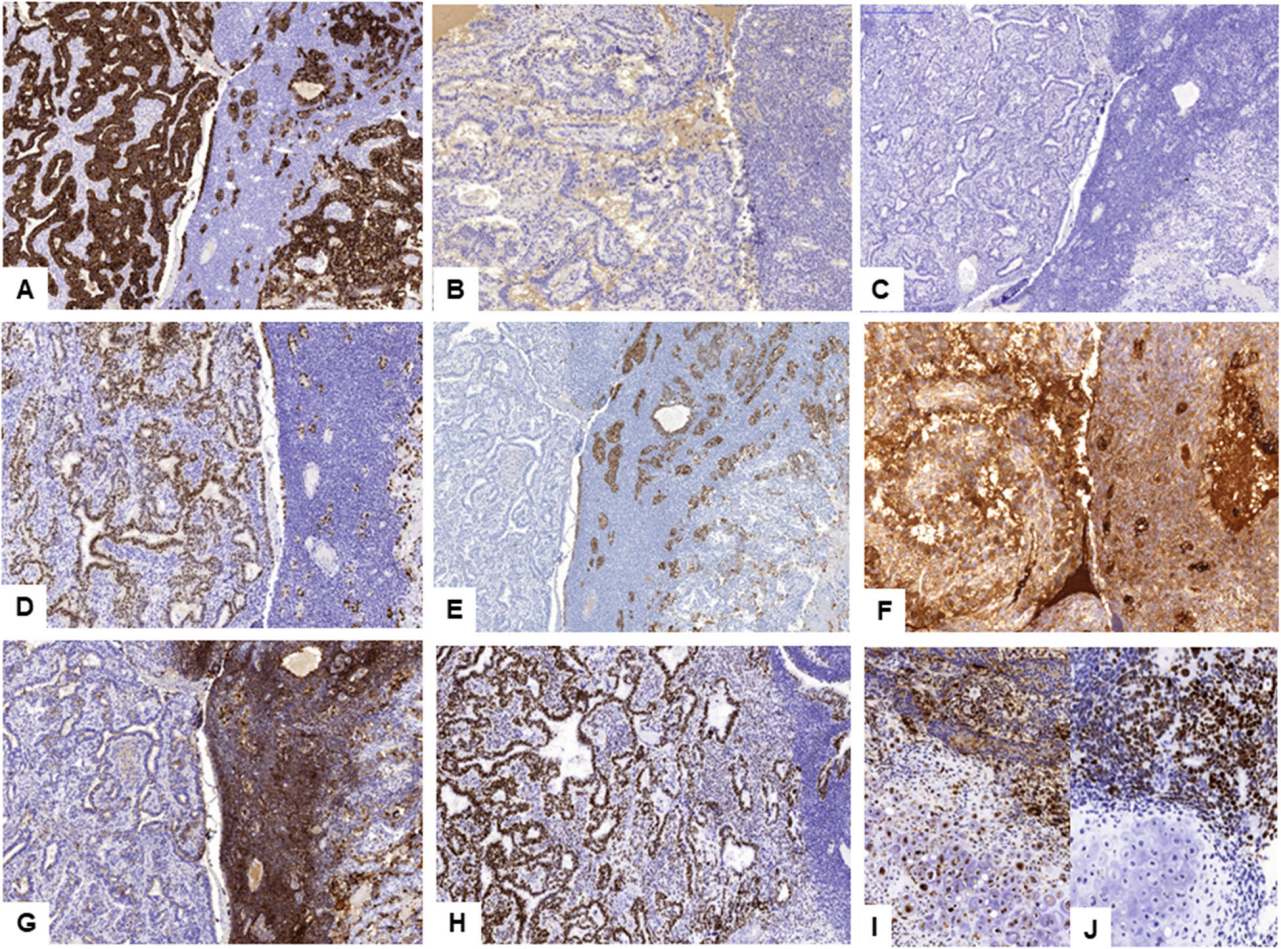
Immunohistochemistry results for the surgical specimen of cystectomy. CK A1/A3 (**a**). EMA (**b**). CK7 (**c**). CDX2 (**d**). HepPar1 (**e**). AFP (**f**). GLP3 (**g**). SALL4 (**h**). GLP3 and SALL4 in sarcomatoid areas with chondroid differentiation (**i-j**)

Immunohistochemistry results are listed in Table 1 and illustrated in Fig. 3. Dual color fluorescent in situ hybridization (FISH) was performed to explore structural or numeric alterations in the chromosome 12. We used the probes 12p13.33 Chr12pTel for the short arm of chromosome 12 (12p), and SureFISH Chr12CEP for the centromere (CEP12) (acquired from Agilent Technologies, CA, USA). According to published methods [5], we calculated the ratio between 12p/CEP12 signals. A ratio above 1.5 was considered indicative of an isochromosome 12 (i12p), while the presence of 3 to 7 pairs of 12p/CEP12 signals in a single nucleus, in 15% or more of the 50 tumor cells evaluated, was considered a polysomic pattern of chromosome 12. In this case, the FISH study showed a polysomic pattern of chromosome 12, but an i12p was not identified.

**Table 1:** RESULTS OF IMMUNOHISTOCHEMISTRY

Antibody	Observation
CK AE1/AE3	Diffusely positive, except in chondroid nodules
CDX2	Positive in glandular areas
Hep Par 1	Positive in hepatoid areas
AFP	Diffusely positive, more intense in glandular and hepatoid areas
GLP3	Diffusely positive, more intense in glandular and hepatoid areas
SALL 4	Diffusely positive, more intense in glandular and hepatoid areas
GATA 3	Negative (positive in the initial urothelial component)
VIM	Positive in small undifferentiated cells
OCT 3/4	Negative
EMA	Negative
CK7	Negative
SYN	Negative

The thoracic mass showed a pure glandular proliferation with morphological and immunohistochemical features similar to those observed in the bladder tumor, so it was considered as a tumoral seeding along the surgical trocar path. After surgery, the patient received chemotherapy cycles with poor response, and several systemic complications. Pulmonary and brain metastasis were identified nine months after cystectomy. The patient died eleven months after diagnosis, and an autopsy was not performed.

## Discussion and conclusions

In this report we present a unique case of YST differentiation arising from a recurrent urothelial carcinoma in a 76-year-old man. This tumor showed a mixture of several histologic patterns, including a sarcomatoid component, which is extremely infrequent.

According to the evidence available, YSTs may arise from benign and malignant somatic lesions in several organs. For example, it is well documented the transformation of benign or malignant ovarian and endometrial tumors into YST [6]. Neoplasms with YST differentiation have also been observed in the sinonasal region, where they have been denominated teratocarcinosarcoma [7], in the stomach, colon, and lung [8]. YST differentiation is not recognized as a histological variant in the current WHO classifications of urothelial carcinoma. However, some reports of urinary neoplasms showing some YST-like features are available in the literature. The majority of these cases have been summarized in a previous publication by Samaratunga et al. [4], which included one urothelial carcinoma, and four adenocarcinomas of the renal pelvis, all exhibiting hepatoid areas and AFP positivity; eight cases of bladder adenocarcinoma with hepatoid areas, and two cases of conventional bladder urothelial carcinoma, all of them showing AFP positivity. Reports of urothelial carcinoma with SALL4 positive areas are also available [9]. Among those cases, additional immunochemistry studies have not been performed to confirm YST differentiation. McNamee et al. proposed the term “somatically derived YSTs” [2] to denominate the spectrum of somatic neoplasms with YST-like features arising most frequently in somatic neoplasms, and suggested unification of the terminology between different sites where such neoplasms occur.

Histologically, our case showed a mixture of diverse patterns, ranging from classical reticular-microcystic to sarcomatoid pattern with chondroid differentiation. Indeed, the most relevant histological characteristic of the somatic neoplasms with YST differentiation is the identification of several patterns, which can be grouped in two categories: the classical ones, which comprise reticular-microcystic, polyvesicular, vitelline, solid and parietal patterns, and the special ones, which comprise glandular, hepatoid and sarcomatoid patterns [1]. The glandular pattern, also referred to as “enteroblastic”, is the most frequently observed among somatic neoplasms with YST differentiation [2], while sarcomatoid pattern is extremely uncommon [10]. In addition to the morphologic features, the diagnosis of YST differentiation requires confirmation with a characteristic immunohistochemistry profile. A majority of these neoplasms shows positivity for AFP, SALL4, and negativity for differentiated epithelial markers, such as CK7 and EMA [2]. CDX2 is usually positive in areas with glandular pattern, while hepatic differentiation markers, such as HepPar 1, may be positive in hepatoid areas. Our case showed the described immunohistochemistry profile, in agreement with the diagnosis of YST differentiation. Moreover, immunohistochemistry performed in previous biopsy specimens, showed similar results in the areas with glandular differentiation (Fig. 1e-h).

We identified genetic alterations in the short arm of chromosome 12, which showed a polysomic pattern, but an i12p was not identified. The polysomic pattern of 12p abnormality has been described previously in germ cell tumors of the central nervous system, although it is of unknown significance [5]. Chromosome 12 abnormalities, either as an i12p or as 12p overrepresentation, are the hallmark cytogenetic alteration of germ cell tumors. These alterations are identified in the majority of gonadal germ cell tumors, with few exceptions [11], and in somatic type malignancies, derived from germ cell tumors. However, the occurrence of chromosome 12 abnormalities is not well established in somatic neoplasms with YST differentiation. While some authors identified i12p and other alterations of chromosome 12 [12], other authors reported cases without i12p, arguing that their results were consistent with the possible somatic origin of the YST component in these neoplasms [10]. The contradictory results may be due to problems in interpretation of the FISH, which is a common assay to identify i12p, but can be difficult to evaluate, and lacks of ideal sensitivity and specificity [13]. Also, the results may be explained by uncertainties regarding the actual origin of the neoplastic cells in somatic tumors with YST differentiation, which may derive from somatic neoplastic cells through a process of retrodifferentiation or neometaplasia, or from a pluripotential embryonic stem/germ cell [2]. Currently, further investigation is required to better characterize genetic alterations in YSTs derived from somatic neoplasms, and to elucidate their actual histogenesis.

Our patient had some clinical characteristics described previously among patients with urinary tumors exhibiting YST features. According to the available reports [4], these tumors are more frequently diagnosed among men of 73 years-old on average, thus differing from primary extragonadal YST that are more common in young patients. Elevated serum Alphafetoprotein (AFP) levels may be also observed among these patients. Regarding our case, these levels were not determined, because this enzyme is not routinely measured in post-surgical controls of adult patients with a bladder carcinoma. However, AFP serum levels should be determined in patients with urothelial neoplasms exhibiting infrequent histological patterns (e.g. glandular or hepatoid), because the elevation of these levels support the diagnosis of YST differentiation. Moreover, these determinations may be used to control the postsurgical evolution, and to detect tumor relapse among these patients [14].

Differential diagnosis included metastatic YST from the testicles, primary extragonadal YST, sarcomatoid urothelial carcinoma, chordoid urothelial carcinoma [15], and carcinoma with enteroblastic or hepatoid differentiation. Metastatic YST and primary extragonadal YST of the bladder are extremely infrequent, and were ruled out after the revision of previous biopsies from the same anatomical location, which showed a primary tumor comprised of conventional urothelial carcinoma admixed with areas of glandular differentiation (Fig. 1a-d). These latter areas were morphologically and histochemically similar to the glandular component of the cystectomy specimen, supporting the diagnosis of urothelial carcinoma with YST differentiation. Sarcomatoid and chordoid urothelial carcinoma were excluded after the identification of areas with classical YST pattern and immunohistochemistry results. Carcinoma with enteroblastic differentiation has been reported in several organs (gastrointestinal, lung, etc) [16], while carcinoma with hepatoid differentiation has been mainly reported in gynecologic tumors [17], and in urinary tract tumors already mentioned [4]. These neoplasms show some histologic and immunohistochemical features, similar to those identified in our case. As previously discussed, according to some authors [2], tumors with enteroblastic and/or hepatoid differentiation could be considered part of the wide spectrum of the so-called SD-YSTs.

In summary, this is a unique case of a bladder urothelial carcinoma with YST differentiation, showing multiple and complex histological patterns. YST differentiation should be suspected in adult patients with extragonadal somatic tumors, also urothelial carcinoma, exhibiting diverse histological patterns, particularly glandular and hepatoid. The diagnosis of YST differentiation in somatic neoplasms requires confirmation with immunohistochemistry results, which necessarily include positivity for AFP, GLP3 and SALL4, and negativity for epithelial markers like CK7 and EMA. Elevated AFP serum levels may support the diagnosis, when this determination is available.

## Supplementary information

**Additional file 1.** CARE checklist.

## Data Availability

All data generated or analyzed during this study are included in this published article.

## Abbreviations

SD-YST: Somatically derived Yolk sac tumor
18F-FDG PET/CT: 2-deoxy-2-[fluorine-18] fluoro- D-glucose integrated with computed tomography
CK AE1/AE3: Cytokeratin AE1-AE3
CDX2: Caudal type homeobox 2
Hep Par 1: Hepatocyte Paraffin 1
AFP: Alphafetoprotein
GLP3: Glypican 3
SALL4: Sal-like protein 4
VIM: Vimentin
OCT 3/4: Octamer-binding transcription factor 3/4
EMA: Epithelial Membrane Antigen
CK7: Cytokeratin 7
SYN: Synaptophysin
CEP12: Centromere of chromosome 12
12p: Short arm of chromosome 12
i12p: Isochromosome 12
